# Transcriptomic analyses show that 24-epibrassinolide (EBR) promotes cold tolerance in cotton seedlings

**DOI:** 10.1371/journal.pone.0245070

**Published:** 2021-02-01

**Authors:** Lingling Dou, Yaru Sun, Shuye Li, Changwei Ge, Qian Shen, Huaizhu Li, Wenbo Wang, Jiayi Mao, Guanghui Xiao, Chaoyou Pang

**Affiliations:** 1 School of Chemistry and Chemical Engineering, Xianyang Normal University, Xianyang, Shaanxi, China; 2 State Key Laboratory of Crop Stress Adaptation and Improvement, School of Life Sciences, Henan University, Kaifeng, Henan, China; 3 State Key Laboratory of Cotton Biology, Institute of Cotton Research, Chinese Academy of Agricultural Sciences, Anyang, Henan, China; 4 College of Life Sciences, Shaanxi Normal University, Xi’an, China; University of Delhi, INDIA

## Abstract

In plants, brassinosteroids (BRs) are a class of steroidal hormones that are involved in numerous physiological responses. However, the function of BRs in cold tolerance in cotton has not been explored. In this study, cotton seedlings were treated with five concentrations (0, 0.05, 0.1, 0.2, 0.5 and 1.0 mg/L) of 24-Epibrassinolide (EBR) at 4°C. We measured the electrolyte leakage, malondialdehyde (MDA) content, proline content, and net photosynthesis rate (Pn) of the seedlings, which showed that EBR treatment increased cold tolerance in cotton in a dose-dependent manner, and that 0.2 mg/L is an optimum concentration for enhancing cold tolerance. The function of EBR in cotton cotyledons was investigated in the control 0 mg/L (Cold+water) and 0.2 mg/L (Cold+EBR) treatments using RNA-Seq. A total of 4,001 differentially expressed genes (DEGs), including 2,591 up-regulated genes and 1,409 down-regulated genes were identified. Gene Ontology (GO) and biochemical pathway enrichment analyses showed that EBR is involved in the genetic information process, secondary metabolism, and also inhibits abscisic acid (ABA) and ethylene (ETH) signal transduction. In this study, physiological experiments showed that EBR can increase cold tolerance in cotton seedlings, and the comprehensive RNA-seq data shed light on the mechanisms through which EBR increases cold tolerance in cotton seedlings.

## 1. Introduction

Cotton is a warm season crop that is sensitive to low temperatures, and it is one of the most important economic crops that is grown around the world because of its valuable textile fiber and edible seed oil. In China, Xinjiang is the main cotton fiber producing area, and cold stress is the major adverse factor that limits cotton production in the Xinjiang cotton-growing region. Cotton seedlings are prone to suffer from the cold stress in late spring, which can seriously affect growth and production [1]. To promote the sustainable development of the cotton industry in Xinjiang, it is very urgent that we improve cotton tolerance to cold stress. At present, there are many studies on the physiological and biochemical effects of cold stress on the yield and quality of cotton [2–4]. However, there are few reports about tolerance to cold damage in cotton seedlings.

In plants, brassinosteroids (BR) are a class of steroidal hormones that are involved in numerous physiological responses, including photomorphogenesis, increase in cell elongation, pollen tube growth, leaf expansion, xylem differentiation, and delayed senescence [5–8]. BRs play an irreplaceable role in plant growth and development, and also play roles in enhancement of resistance to adverse conditions by reducing the damage caused by stress, thus maintaining plant growth and development and avoiding crop yield losses [8].

BRs are also involved in plant biotic and abiotic stress resistance. Under drought stress, exogenously applied BR could alleviate drought-induced oxidative stress, reduce malondialdehyde (MDA) content, and enhance tolerance to drought in maize [9]. BR treatment also increased lipid production and tolerance of *Chlorella* cells to high-temperature stress [10]. Excessive copper can be toxic to sunflower, and application of BR can alleviate the toxic effect of copper [11]. In *Arabidopsis thaliana*, endoplasmic reticulum- (ER)-associated protein degradation (ERAD) is involved in BR-mediated tolerance to salt stress [12]. BRs function in mediating the effects of *Verticillium dahliae* (Vd) toxin in upland cotton [13]. BR can increase the net photosynthetic rate (Pn) which is accompanied by significant increase in photosynthesis during cold stress [6]. However, there is little information available about how BR functions in cold tolerance in cotton.

We used next-generation high-throughput nucleotide sequencing to obtain global gene expression profiles and provide a precise measurement of transcript levels [14]. In this study, the effect of EBR on cold tolerance in cotton was elucidated: (I) exogenous application of EBR enhanced cold tolerance in cotton seedlings in a dose-dependent manner, and 0.2 mg/L was found to be the optimal concentration for cold stress tolerance. (II) The differentially-expressed genes (DEGs) were significantly enriched in three categories of biochemical pathways: (i) the DEGs were significantly enriched in gossypol biosynthesis and glutathione metabolism in response to cold treatment; (ii) EBR promotes genetic information processing under cold stress; and (iii) EBR increased cold tolerance in cotton seedlings by down-regulating ETH and ABA signal transduction.

## 2. Materials and methods

### 2.1 Plant growth and treatments

Seeds of the cotton cultivar ‘Lumianyan 28’ were sterilized with 15% sodium hypochlorite for 15 minutes, then rinsed five times and immersed in sterilized water for 12 hours before sowing. The seeds were planted under controlled conditions (28°C day/20°C night, 16 h photoperiod, 60% humidity) in a greenhouse at the Cotton Research Institute of the Chinese Academy of Agricultural Sciences (CAAS), Anyang, Henan. Two weeks after emergence, the cotyledons were fully flattened and plants with the same growth were divided into 18 groups; each group contained nine plants, and they were sprayed with 24-epibrassinolide (EBR) at five concentrations including 0 mg/L (CK, ddH_2_O), 0.05 mg/L (T1), 0.1 mg/L (T2), 0.2 mg/L (T3), 0.5 mg/L (T4), and 1.0 mg/L (T5) to control BR levels in cotton cotyledons at 4°C for 24 h [9] with the other growth parameters (28°C day/20°C night, 16 h photoperiod, 60% humidity) unchanged [15]. Samples were collected and immediately frozen in liquid nitrogen and then stored at -80°C.

### 2.2 Measurement of physiological traits in the cotyledons

The net photosynthetic rate (Pn) was measured in the cotyledons in nine pots using a Li6400 photosynthesis system (Li COR Inc., NE, USA) with a light intensity of 1800 μmol (photon) m^-2^ s^-1^. The CO_2_ level was 400 mol in an open gas-exchange system [16].

The content of MDA was measured by the thiobarbituric acid (TBA) method [17]. The proline content was measured using the ninhydrin reaction method of Bates et al. [18].

The cotyledons were rinsed with distilled water three times and then five disc punches (0.75 cm^2^) were sampled for each treatment group. The discs were incubated at 25°C for 10 hours in the dark. The electrical conductivity of the solution (R1) of all samples was then measured using a conductivity meter (Mettler Toledo, USA), after which the solution was boiled for 30 min and the electrical conductivity was measured for the second time (R2) after the samples were equilibrated at 25°C. The relative electrical conductivity (REC) was calculated as REC = R1/R2×100% [19]. The REC is an indicator of cold injury; higher conductivity shows a higher degree of stress [20].

All the experiments were repeated three times with nine plants per treatment. The data for the physiological traits were displayed as the means of the independent biological replicates± standard deviation (SD). Analysis of variance was performed using SPSS statistics 16 software. Significant differences were determined at the 5% and 1% levels of significance and lower-case letters were used to indicate the p-values [21, 22].

### 2.3 RNA extraction and RNA-seq

Total RNA was extracted from each sample using the E.Z.N.A.^®^ Plant RNA Kit (Omega Bio-tek, USA), and the RNA integrity and concentration were estimated by agarose gel electrophoresis and with an Agilent 2100 bioanalyzer, respectively, as described previously [23]. Following total RNA extraction, the mRNA was enriched using oligo-dT magnetic beads, and the enriched mRNA was fragmented and reverse transcribed into cDNA using random primers. The second-strand cDNA was synthesized using NEBNext^®^ Second Strand Synthesis Enzyme Mix (New England BioLabs, Ipswich, MA), the cDNA was purified using 1.8X Agencourt AMPure XP Beads (Beckman, USA) and then ligated with the Illumina sequencing adapter. The RNA sequencing was performed on an Illumina HiSeq 2500 at Gene Denovo Biotechnology Co. (Guangzhou, China) as previously described [24]. The sequencing data from this study has been deposited under SRA accession PRJNA640886.

### 2.4 Data processing

To obtain high-quality clean reads, the raw sequencing data was filtered by the following criteria: Reads consisting of adapter sequences were removed, as were reads containing >10% unknown bases (N) and low-quality reads in which the number of bases with quality scores ≤20 accounted for >50%. Reads containing only poly-A were removed, and the reads that mapped to rRNA were also removed. We used TopHat2, a transcriptome data comparison software [25], to map the HQ clean reads to the *G*. *hirsutum* L. reference genome (NAU, version 1.1) [26], and Cufflinks was used to assemble the reads into full-length transcripts [27]. The fragments per kilobase of transcript per million mapped reads (FPKM) method were used to calculate relative gene expression values [28]. Pearson correlation coefficients were used to explore the relationships between samples and were calculated using R statistical software [29]. Based on the FPKM expression values, the edgeR package was used to identify the DEGs. DEGs were identified using the following thresholds: the absolute value of log_2_(ratio of two FPKM values) >1, the p value and false discovery rate (FDR) are <0.05) [30].

### 2.5 GO (Gene Ontology) and KEGG (Kyoto Encyclopedia of Genes and Genomes) analyses

After the DEGs were identified, we conducted GO term enrichment (http://amigo.geneontology.org) and categorized the DEGs using AgriGO software [31]. GO enrichment analysis provides all GO terms that are significantly enriched in the DEGs compared to the genome background, and filters the DEGs that correspond to biological functions. All DEGs were mapped to GO terms in the Gene Ontology database (http://www.geneontology.org/), gene numbers were calculated for every term, and significantly enriched GO terms in the DEGs compared to the genome background and the p-value were defined by a hypergeometric test. The p-values were corrected to control the FDR, taking FDR ≤0.05 as a threshold [32]. The KEGG orthology-based Annotation System (KOBAS) was used to identify biochemical pathways involved in EBR-increased cold tolerance, and both the p-value and FDR of the KEGG pathways were calculated [33]. GO terms and KEGG pathways meeting the p-value and with FDR <0.05 were defined as significantly enriched GO-terms and pathways in the DEGs, respectively [34].

## 3. Results

### 3.1 EBR alleviates cold stress in cotton seedlings on a physiological level in a dose-dependent manner

**Fig 1:**
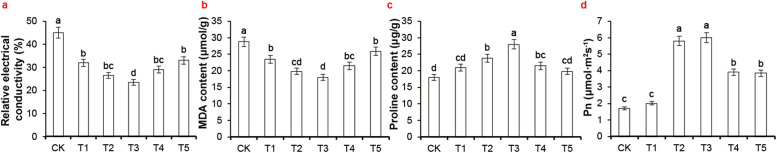
The effects of different EBR treatments on the relative conductivity, MDA content, proline content, and net photosynthetic rate (Pn) in cotton seedlings. Note, the concentrations of EBR in the six treatments—0, 0.05, 0.1, 0.2, 0.5, and 1.0 mg/L- are indicted as as CK, T1, T2, T3, T4, and T5, respectively on the x-axis. Letters above the bar indicate significant differences (p = 0.05), and error bars represent the standard errors.

To measure the effect of EBR on cotton cotyledons under cold stress (4°C), a concentration range (0, 0.05, 0.1, 0.2, 0.5 and 1.0 mg/L) was used to determine an optimum EBR concentration for the subsequent experiments. Considering that the main function of cotyledons is photosynthesis, we measured the net photosynthetic rate (Pn). Cold stress first damages cell membranes and MDA is a product of lipid peroxidation, and increasing levels of MDA can further damage the cell membrane. Therefore the MDA content can reflect the relative degree of stress [35, 36]. Proline functions as an important osmoprotectant in the plant response to adverse conditions; a higher proline content indicates better tolerance to cold stress [37]. The relative electrolyte leakage (REC), Pn, MDA, and proline contents were measured after the seedlings were sprayed with EBR at 4°C for 24 hours. Under cold stress conditions, the REC and MDA content in the CK (0 mg/L EBR) were the highest, but were the lowest in the T3 (0.2 mg/L) treatment (Fig 1a and 1b); the proline content and Pn in the CK were the lowest and were the highest in T3 (Fig 1c and 1d). Above all, the EBR treatments increased Pn and proline content, and decreased the REC and MDA contents in the cotton seedlings; also, EBR increased cotton cold (4°C) tolerance in a dose-dependent manner, and 0.2 mg/L was found to be an optimum concentration for spraying cotton seedlings to enhance cold tolerance.

### 3.2 High-quality transcriptome data obtained by Illumina sequencing

**Fig 2:**
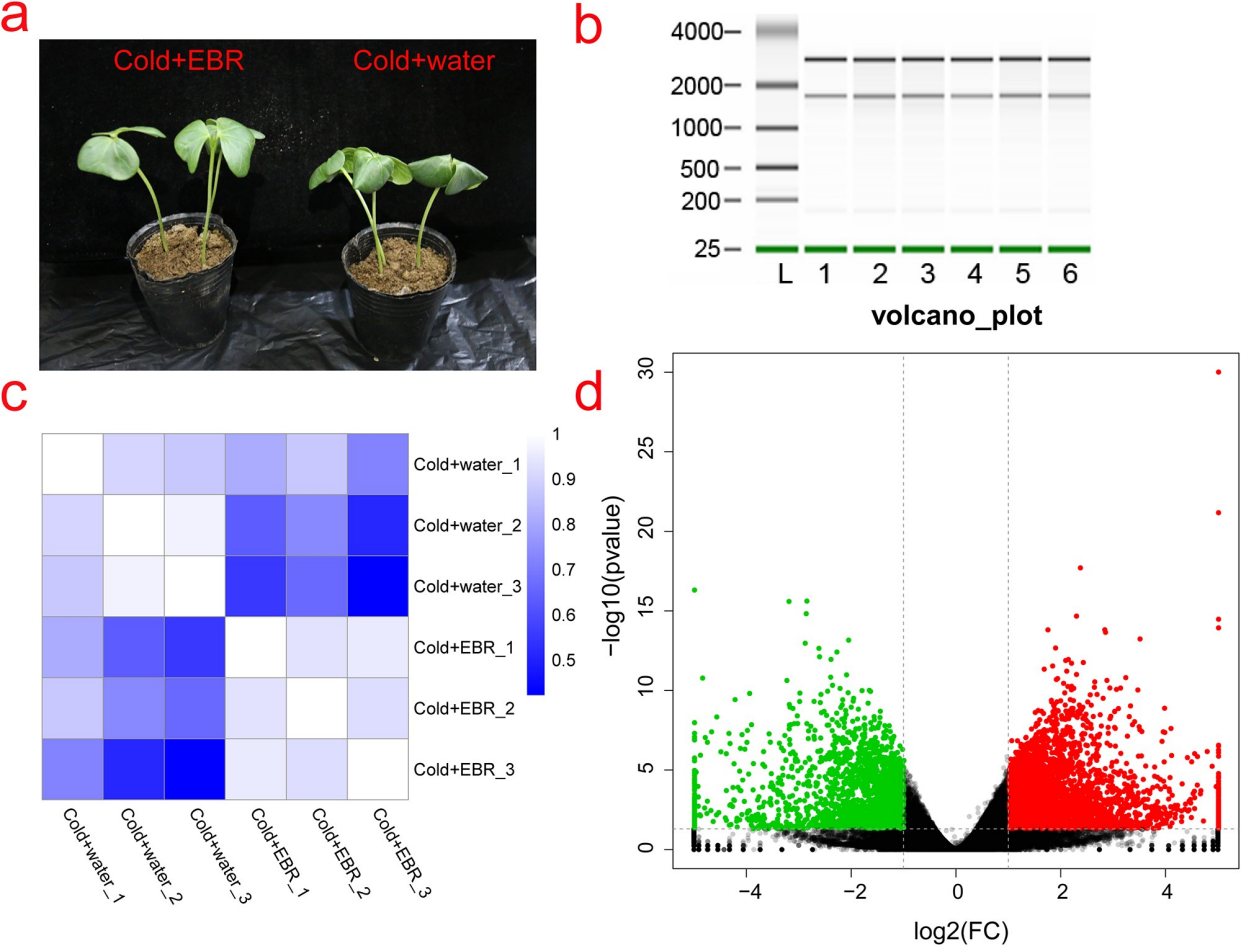
Statistical analysis of the differentially expressed genes. **a**. The phenotypes of cotton cotyledons with- or without EBR treatments after exposure to cold stress for 24 h. **b**. Total RNA extracted from ‘Lumianyan 28’ leaves. The numbers on the left represent the DNA marker ladder lengths (base pair, bp). L, DNA ladder. The numbers 1–6 represent RNA samples from the Cold+water_1, Cold+water_2, Cold+water_3, Cold+EBR_1, Cold+EBR_2, and Cold+EBR_3 treatments, respectively. **c**. A heatmap of Pearson correlation coefficients from comparisons of the Cold+water and Cold+EBR samples (three replicates of each). The color bar from blue to red indicates the correlation coefficients from 1 to 0. **d**. Volcano plot showing the DEGs in the two different libraries. The q<0.05 was used as the threshold to determine the significance of the DEGs. Red dots represent up-regulated genes, green dots represent down-regulated genes, and the black dots indicate transcripts that did not change significantly between the Cold+water vs. Cold+EBR treatment libraries.

In cotton seedlings exposed to cold stress (4°C) for 24 hours, the plants (Cold+EBR) sprayed with 0.2 mg/L EBR grew well, while the control plants (Cold+water) only treated with H_2_O showing wilting (Fig 2a), which indicated that 0.2 mg/L EBR can increase the cold tolerance of cotton seedlings. To obtain a global picture of how EBR functions in cold cotton tolerance, RNA extracted from the cotyledons of the Cold+EBR (0.2 mg/L) and Cold+water treatments were sequenced, respectively. RNA extracted from the six samples showed three clean bands (Fig 2b) and the mRNA was enriched from the total RNA to construct libraries for RNA sequencing; the samples were labeled Cold+water_1, Cold+water_2, Cold+water_3, Cold+EBR_1, Cold+EBR_2, and Cold+EBR_3. The raw data was filtered, and the high quality (HQ) clean reads from the six libraries ranged from 6,034,895,849 bp to 8,708,230,676 bp. In the HQ clean read data, the percentages of reads with quality scores of Q20 and Q30 were 96.28% and 94.61%, respectively. The average GC content of the HQ clean reads was 46.87% (Table 1). The HQ clean reads from each library were aligned to the *G*. *hirsutum* L. reference genome (NAU, version 1.1) [26]. The mapped proportion of the HQ clean reads in the six transcriptome libraries ranged from 90.54% to 94.36%, and the uniquely-mapped reads accounted for 84.05% to 87.90% (Table 1). The proportions of HQ reads and mapped reads indicate that the RNA-seq was well performed and was of high quality.

**Table 1:** Summary of RNA-seq read assembly.

samples	Raw Reads	Clean Reads	Q20(%)	Q30(%)	GC(%)	Unique Mapped Reads
Cold+water_1	9,087,681,300	8,708,230,676	8,552,002,590 (98.21%)	8,228,707,273 (94.49%)	4,093,053,367 (47.00%)	51,378,347 (87.90%)
Cold+water_2	8,187,926,400	7,848,638,271	7,712,655,754 (98.27%)	7,428,768,598 (94.65%)	3,704,220,342 (47.20%)	46,127,325 (87.79%)
Cold+water_3	6,563,257,200	6,034,895,849	5,810,546,470 (96.28%)	5,402,624,814 (89.52%)	2,881,473,267 (47.75%)	34,821,154 (84.05%)
Cold+EBR_1	8,092,116,900	7,778,207,520	7,644,217,507 (98.28%)	7,369,526,818 (94.75%)	3,586,860,441 (46.11%)	46,038,053 (87.83%)
Cold+EBR_2	8,320,120,200	7,982,325,576	7,841,013,843 (98.23%)	7,553,001,096 (94.62%)	3,712,727,215 (46.51%)	47,359,809 (87.72%)
Cold+EBR_3	8,634,929,700	8,269,599,826	8,124,580,366 (98.25%)	7,823,653,210 (94.61%)	3,850,116,449 (46.56%)	48,852,593 (87.70%)

### 3.3 Statistical analysis of the differentially expressed genes

To further analyze the reproducibility among biological replicates of the RNA-seq data, the Pearson correlation coefficients were calculated. In Fig 2c, the correlations between the biological replicates were good, with values ranging from 0.89 to 0.97. However, the correlations between samples out of the biological replicates varied from 0.42 to 0.87. These results show that the experiments were performed with high reliability and reproducibility for the biological replicates and the sequencing method. To illustrate the functions of genes involved in EBR-mediated cotton cold tolerance, the DEGs were identified using the criteria in which both the FDR and p-value were <0.05. Compared with the control treatment (Cold+water), there were 2,591 up-regulated genes and 1,409 down-regulated genes (Fig 2d). These results suggest that EBR had a marked effect on the transcription of a subset of genes responsible for cold stress in cotton seedlings.

### 3.4 GO annotation of the DEGs

**Fig 3:**
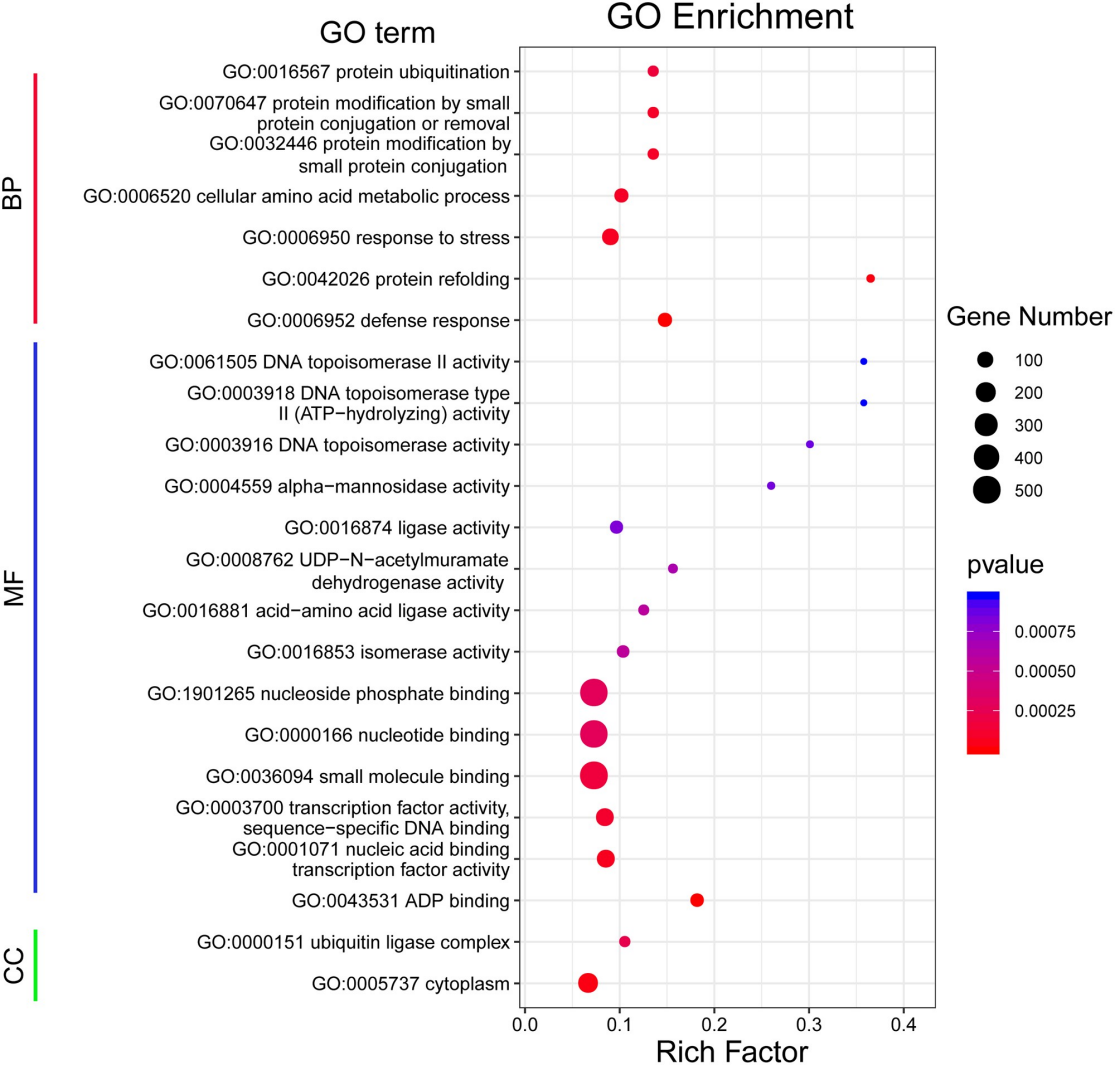
GO enrichment of DEGs identified between the Cold+water vs. Cold+EBR treatments. The major Gene Ontology (GO) categories are shown to the left. The Y-axis indicates the enriched GO terms in the BP, MF, and CC GO categories. The X-axis shows the Rich factor. A high corrected p-value is shown in blue, and low corrected p-values are shown in red (corrected p-value <0.05). BP, biological process; MF, molecular function; CC, cellular component.

To illustrate the global expression profiles of the 4,001 DEGs and reveal major functional categories, we performed GO enrichment analysis using AgriGO. We identified a total of 23 significantly enriched GO terms with both p-value and FDR ≤ 0.05, in the three major GO categories, including two ‘cellular component’ terms, seven ‘biological process’ terms and 14 ‘molecular function’ terms (Fig 3, S1 Table). Among the ‘biological process’ terms, four are involved in protein post-translational modification (GO:0016567, GO:0070647, GO:0032446, and GO:0042026), two GO terms (GO:0006952 defense response) and (GO:0006950 response to stress) are plant stress-related. For the ‘molecular function’ terms, seven are involved in the regulation of gene transcription including (GO:0061505, GO:0003918, GO:0003916, GO:1901265, GO:0000166, GO:0003700, and GO:0001071) and others are related to enzyme activity. For the ‘cellular component’ GO terms, the most significantly overrepresented terms are ‘ubiquitin ligase complex’ (GO:0000151) and ‘cytoplasm’ (GO:0005737). In summary, the GO annotation showed that the DEGs of Cold+water and Cold+EBR were primarily associated with gene transcription and protein post-translational modification.

### 3.5 KEGG pathway analysis of the DEGs

**Fig 4:**
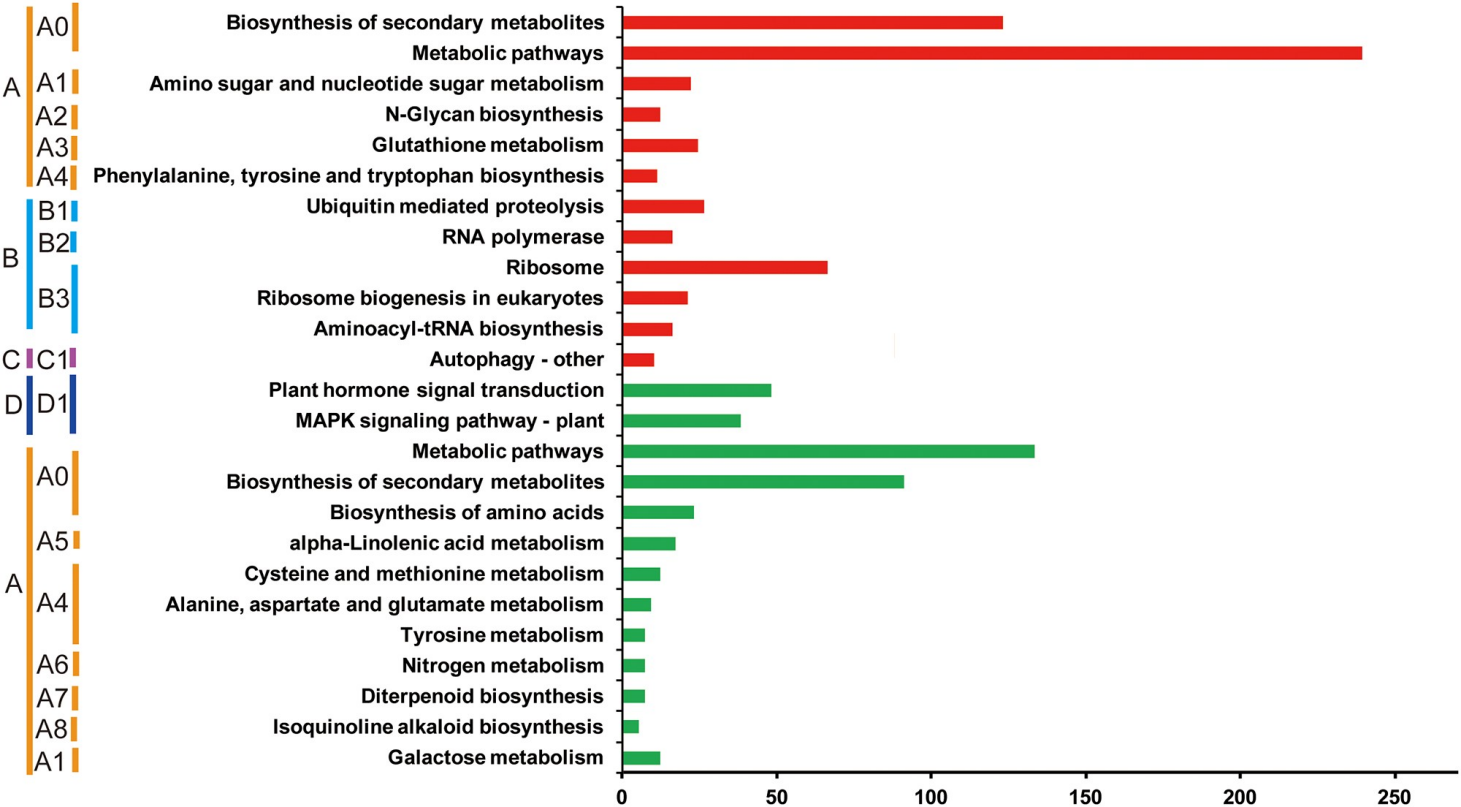
KEGG (Kyoto Encyclopedia of Genes and Genomes) pathway enrichment of DEGs between the Cold+water and Cold+EBR treatments. The Y-axis shows the enriched KEGG terms. The X-axis shows the input gene numbers. The red and green bars indicate the up- and down-regulated DEGs in each enriched KEGG pathway, respectively. On the left, the letter A and A+numbers indicate metabolism and its subcategories. A0, Global and overview maps; A1, Carbohydrate metabolism; A2, Glycan biosynthesis and metabolism; A3, Metabolism of other amino acids; A4, Amino acid metabolism; A5, Lipid metabolism; A6, Energy metabolism; A7, Metabolism of terpenoids and polyketides; A8, Biosynthesis of other secondary metabolites. The letter B and B+number indicate genetic information processing and its subcategories. B1, Folding, sorting and degradation; B2, Transcription; B3, aminoacyl-tRNA biosynthesis. The letter C and C+numbers indicate Cellular Processes and its subcategories, C1, Transport and catabolism. The letter D and D+number indicates environmental information processing and its subcategories, D1, Signal transduction.

To further analyze the biochemical pathways regulated by EBR, the KEGG pathway enrichment analysis of the up- and down-regulated DEGs was performed by KOBAS. The 2,591 up-regulated genes mapped to 121 KEGG pathways, and among them, 12 KEGG pathways were significantly enriched, with corrected p-values ≤0.05 (S2 Table). The 1,409 down-regulated genes mapped to 91 KEGG pathways, and of these, 13 were significantly enriched (S3 Table). Both the up- and down-regulated DEGs were significantly enriched in the biosynthesis of secondary metabolites such as carotenoids, phenylpropanoids, sesquiterpenoids, triterpenoids, and delta- cadinene (Fig 4, S2 and S3 Tables). The up-regulated KEGG pathways mainly included ‘aminoacyl-tRNA biosynthesis’, ‘ribosome’, ‘ribosome biogenesis’, ‘RNA polymerase’, and ‘ubiquitin mediated proteolysis’ (Fig 4). The down-regulated DEGs were significantly enriched in signal transduction, including genes involved in the MAPK signaling pathway and plant hormone signal transduction (Fig 4).

### 3.6 EBR treatment down regulates the gossypol biosynthesis pathway in cotyledons exposed to cold stress

**Fig 5:**
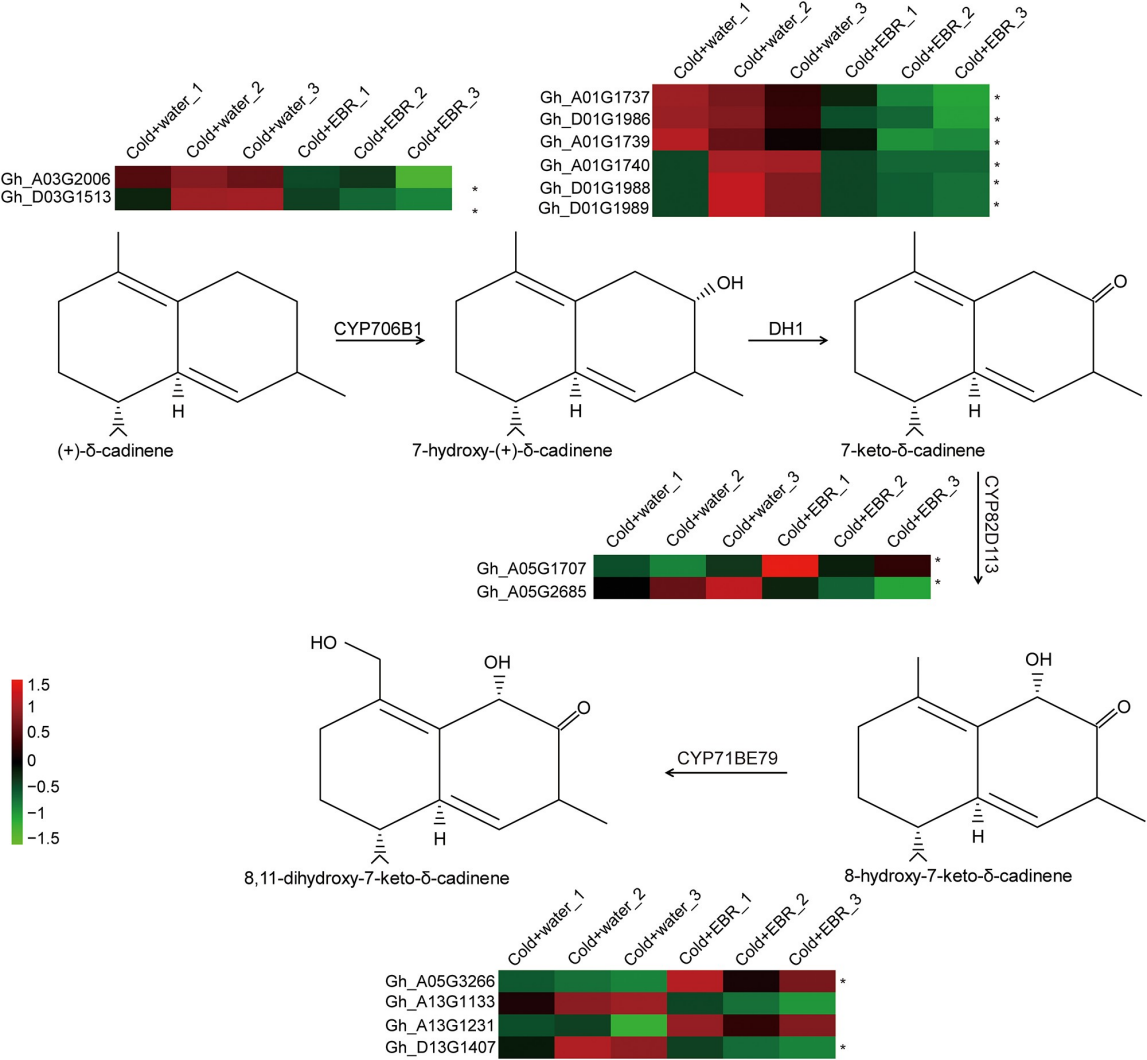
Genes encoding enzymes involved in the gossypol pathway and their expression. Solid arrows indicate identified reactions, the enzymatic reactions found in the RNA-seq data. The FPKM values were used to draw the heatmaps of the three replicates of the Cold+water and Cold+EBR treatments using the online Omicshare tools (https://www.omicshare.com/tools). The colored boxes show the relative gene expression levels from low (green) to high (red). *, indicates genes involved in the gossypol biosynthesis pathway that were identified in previous studies [38].

Considering that both the up- and down-regulated DEGs were significantly enriched in secondary metabolite biosynthesis genes, we further analyzed the related genes. Gossypol is one of the most important secondary metabolites in cotton and, interestingly, we identified a successive gossypol biosynthesis pathway [38]. As shown in Fig 5, two cytochrome P450 (*CYP706B1*) genes are involved in adding hydroxyl groups to (+)-δ-cadinene to form 7-hydroxy-(+)-δ-cadinene; six genes encode alcohol dehydrogenase (DH1) which function in synthesis of 7-keto-δ-cadinene; and two cytochrome P450s (two *CYP82D113* and four *CYP71BE79* genes) are involved in catalyzing 8-hydroxy-7-keto-δ-cadinene and 8,11-dihydroxy-7- keto-δ-cadinene, respectively. Based on the relative gene expression values between the Cold+EBR and Cold+water samples, we concluded that EBR treatment down-regulated gossypol biosynthesis in cotton cotyledons in four successive pathways that included the genes *CYP706B1* (*Gh_A03G2006* and *Gh_D03G1513*), *CYP706B1* (*Gh_A03G2006* and *Gh_D03G1513*), and *DH1* (*Gh_A01G1737*, *Gh_D01G1986*, *Gh_A01G1739*, *Gh_A01G1740*, *Gh_D01G1988* and *Gh_D01G1989*) under conditions of cold stress.

### 3.7 The up-regulated DEGs are significantly enriched in glutathione metabolism genes

**Fig 6:**
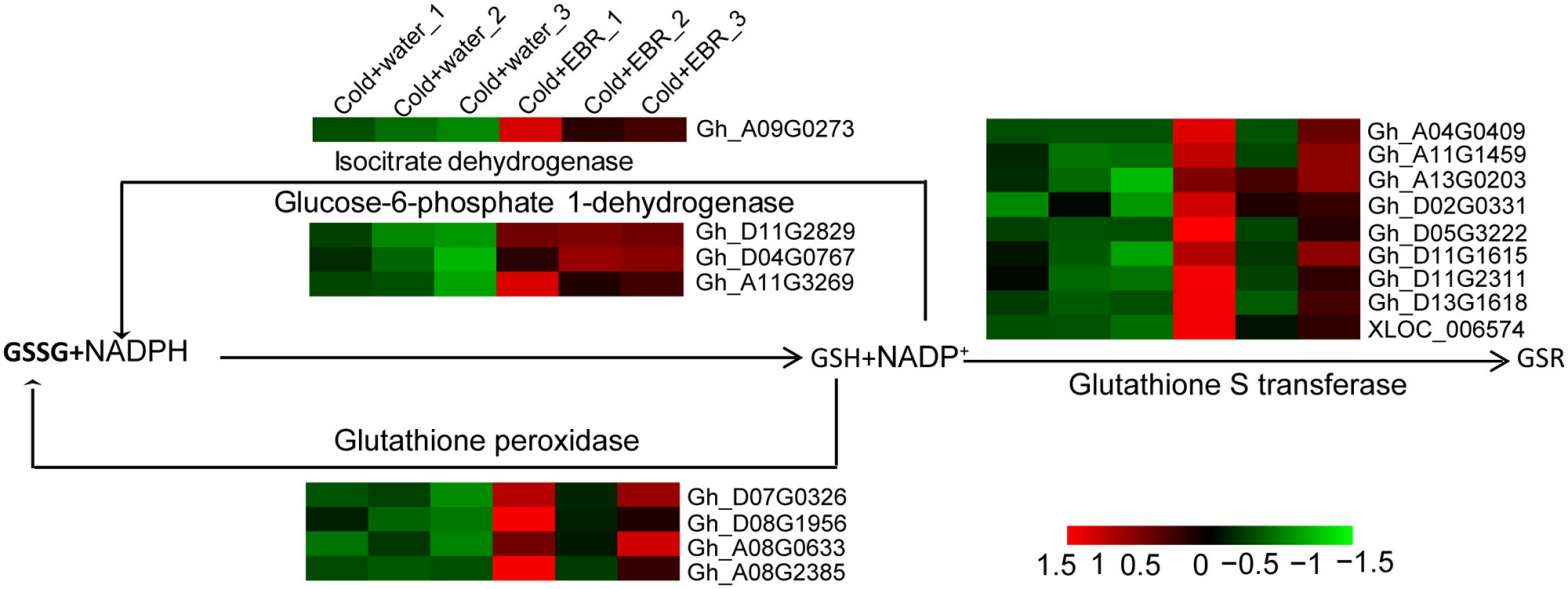
Transcriptional changes in genes involved in glutathione metabolism in cotton cotyledons identified by RNA-seq. The colored squares near the arrows indicate the relative expression of the corresponding homologous genes in the Cold+water and Cold+EBR treatments. GSSG, glutathione disulfide. GSH, glutathione.

Glutathione (GSH) is a tripeptide composed of glutamate, cysteine, and glycine, that can clear free radicals from plant cells and thus eliminate cold stress [39]. Glutathione peroxidase (GPX) and glutathione S-transferase (GST) are two major enzymes involved in glutathione metabolism. Glutathione peroxidase (GPX) oxidizes GSH to glutathione disulfide (GSSG). GST catalyzes the conjugation of GSH to a variety of other compounds via its sulfhydryl groups to reduce free radicals that harm the plant. In Fig 6, expression of four *GPX* and nine *GST* genes was up-regulated by EBR in cotton seedlings exposed to cold stress. Furthermore, genes encoding the enzymes isocitrate dehydrogenase (Gh_A09G0273) and glucose-6-phosphate 1-dehydrogenase (Gh_D11G2829, Gh_D04G0767 and Gh_A11G3269) that promote conversion of oxidized nicotinamide adenine dinucleotide phosphate (NADP^+^) to NADPH were also up-regulated by EBR under cold stress (Fig 6, S4 Table). The up-regulated DEGs were significantly enriched in the glutathione metabolism process, which indicates that glutathione metabolism plays an important role in EBR-alleviated cold stress in cotton cotyledons.

### 3.8 EBR positively regulates photosynthesis-related genes under cold stress conditions

For cotton seedlings, the most important function of the cotyledon is to participate in photosynthesis and provide fixed carbon for plant growth. Exogenous application of EBR can promote the tolerance of cotton seedlings to cold stress. Therefore, it is necessary to analyze the expression patterns of photosynthesis-related genes between the Cold+water and Cold+EBR treatments. Combing the GO and KEGG enrichment analyses, there are 14 photosynthesis-related genes that were up-regulated by EBR in response to cold stress. In Fig 1, compared to the Cold+water group, Pn increased significantly in the Cold+EBR group. The RNA-seq data showed that some photosynthesis-related genes that are involved in the electron transport chain during photosynthesis were up-regulated. In Table 2, there are five genes (*Gh_A13G1559*, *Gh_D13G1896*, *Gh_A10G0605*, *Gh_D10G1053*, and *Gh_D04G0584*) that encode PsbP and one gene (*Gh_D13G1497*) that encodes PsbQ; both PsbP and PsbQ play important roles in the lumenal oxygen-evolving activity of the photosystem II complex [40]. Three genes (*Gh_Sca006756G05*, *Gh_A07G0818*, and *Gh_D07G0878*) participate in photosystem I. *Gh_A12G0547* encodes a leaf ferredoxin-NADP^+^-oxidoreductase 1 (LFNR1), Gh_A05G3061 encodes photosynthetic NDH subcomplex L 1 (PPL2), and both proteins are responsible for the transfer of photosynthetic electrons. *Gh_Sca035444G01* encodes ATP synthase gamma chain (ATPG) which participates in proton-driven ATP synthesis. The up-regulated photosynthesis-related DEGs indicate that BRs may have a positive function in regulating the photosynthesis process under cold stress conditions.

**Table 2:** Photosynthesis-related DEGs identified between the Cold+water and Cold+EBR treatments.

Gene ID	log2(FC)	Pvalue	FDR	Symbol	Description
Gh_A12G1258	1.118901	0.001495	0.021562	PB27A	Photosystem II repair protein PSB27-H1
Gh_D12G1385	1.175268	8.83E-05	0.002911	PB27A	Photosystem II repair protein PSB27-H1
Gh_Sca006756G05	2.212404	5.2E-05	0.002006	PSAB	Photosystem I P700 chlorophyll a apoprotein A2
Gh_A07G0818	2.47029	3.18E-09	1.52E-06	PSAL	Photosystem I subunit l
Gh_D07G0878	1.624548	3.42E-06	0.000273	PSAL	Photosystem I reaction center subunit XI
Gh_A05G3061	1.942967	9.36E-07	0.000105	PPL2	Photosynthetic NDH subcomplex L 1
Gh_Sca035444G01	2.552162	1.93E-10	1.87E-07	ATPG	ATP synthase gamma chain
Gh_A12G0547	4.653442	0.000322	0.007263	LFNR1	Ferredoxin-NADP(+)-oxidoreductase 1
Gh_A13G1559	1.778532	0.000103	0.003252	PPD1	PsbP domain-containing protein 1
Gh_D13G1896	1.696496	8.61E-05	0.002852	PPD1	PsbP domain-containing protein 1
Gh_A10G0605	2.412863	9.84E-06	0.000602	PPD2	PsbP domain-containing protein 2
Gh_D10G1053	1.472251	0.000897	0.015122	PPD2	PsbP domain-containing protein 2
Gh_D04G0584	1.41605	0.000975	0.016064	PPL2	PsbP-like protein 2
Gh_D13G1497	1.287483	5.5E-06	0.000388	PQL2	PsbQ-like protein 2

Note: Photosystem II (PSII)1 is a pigment-protein complex, which consists of at least 25 different protein subunits, at present denoted PsbA-Z according to the genes that encode them. PsbQ plays an important role in the lumenal oxygen-evolving activity of PSII from higher plants and green algae. PsbP, is a membrane-extrinsic subunit of the water-oxidizing complex PSII.

### 3.9 EBR promotes transcription and translation under cold stress conditions

KEGG pathway enrichment analysis showed that the up-regulated DEGs are significantly enriched in six genetic information processing pathways including ‘RNA polymerase’, ‘ribosome’, ‘ribosome biogenesis in eukaryotes’, ‘aminoacyl-tRNA biosynthesis’, ‘ubiquitin mediated proteolysis’, and ‘autophagy’ (Fig 4). These results indicate that EBR treatment under cold stress increased gene transcription, translation, and posttranslational modification.

**Fig 7:**
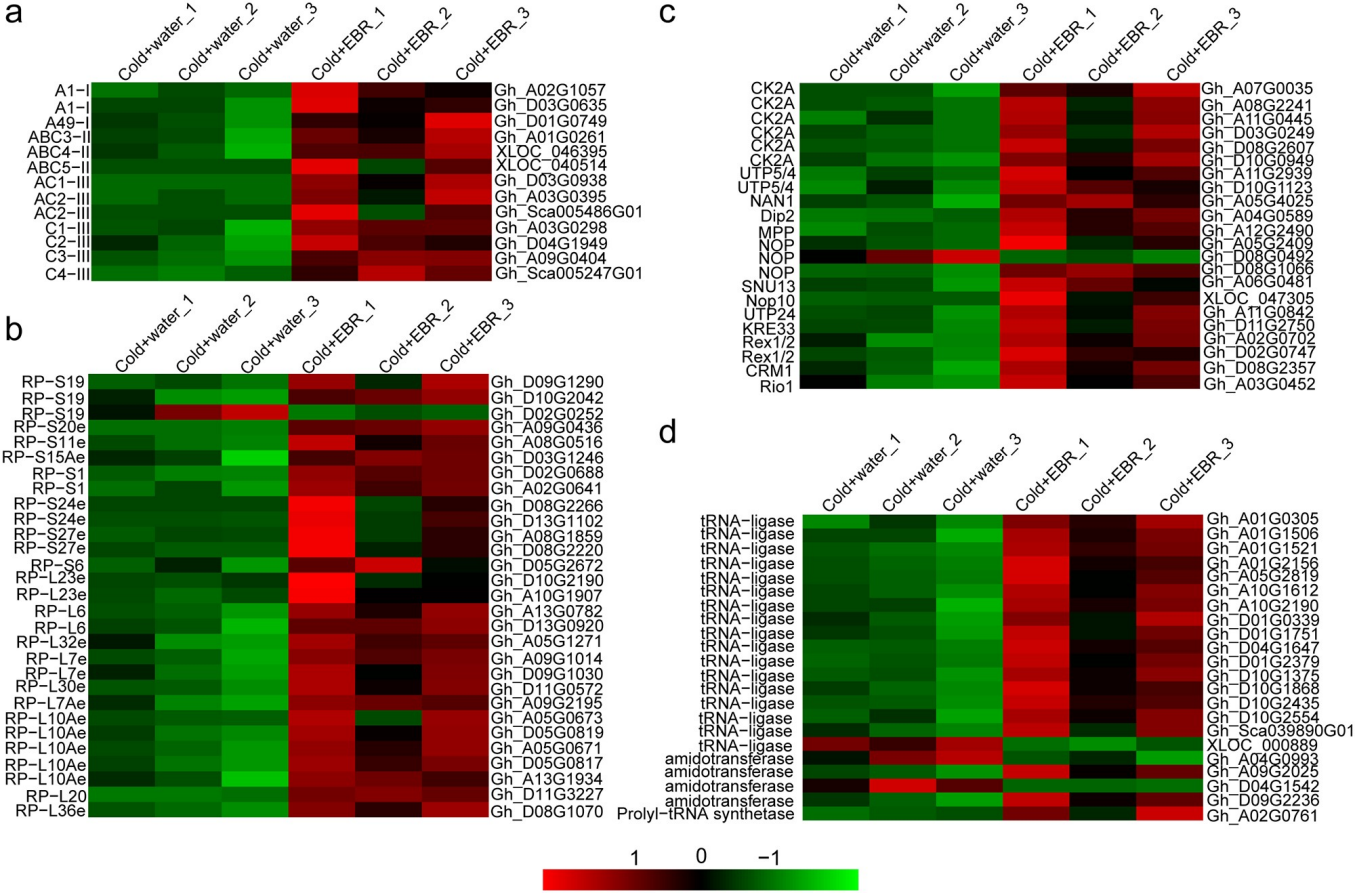
Up-regulated DEGs involved in the transcription and translation processes in cotton cotyledons treated with EBR under cold stress. **a**. DEGs encoding subunits of RNA Pol I, II, and III. **b**. Ribosomal subunit-related DEGs. **c**. Ribosome biogenesis-related DEGs. **d**. tRNA biosynthesis-related DEGs. The colored boxes show the relative gene expression levels from low (green) to high (red). The annotations and gene names are shown on the left and right sides of the heatmaps, respectively.

RNA polymerase binds to the promoter sequence to initiate transcription [41]. Eukaryotic polymerase contains three kinds of polymerases, including polymerase I (Pol I), Pol II, and Pol III. There are 13 up-regulated DEGs involved in the Pol components (Fig 7a and S5 Table), including subunits common to Pol I, II, and III (ABC3, ABC4, and ABC5), core subunits of Pol III (C1, C2, AC1, and AC2), Pol III-specific subunits (C3 and C4), the core subunits of Pol I (A1, AC1, and AC2), and Pol I-specific subunits (A49).

The ribosome is the cellular factory for protein synthesis; biogenesis involves the production and assembly of rRNAs and ribosomal proteins [42]. There were 82 up-regulated DEGs involved in ribosomal components and ribosomal biogenesis (the 40S and 60S subunits) in eukaryotes that were extensively changed in cotton seedlings treated with EBR under cold stress. The ribosomes are composed of large and small subunits. There are 16 up-regulated genes involved in the large subunit proteins (L10e, L20, L23e, L30e, L32e, L36e, L6, L7Ae, and L7e) and 13 genes involved in the small subunit proteins (S1, S11e, S15Ae, S9, S20e, S24e, S27e, and S6) (Fig 7b, S5 Table). There were 24 up-regulated gene homologs which are involved in the 90S pre-ribosome (CK2A, UTP5, UTP4, NAN1, UTP18, UTP21, Dip2, and MPP10), rRNA modification (Nop10, NOP1, and SUN13), cleavages (UTP24, KRE33, Rex1/2, and Nug1/2), and export (CRM1). In the translation process, tRNAs are responsible for transporting the required amino acids to the ribosomes (Fig 7c and S5 Table). We identified 22 up-regulated DEGs involved in tRNA biosynthesis (Fig 7d, S5 Table), which involve aminoacyl-tRNA-ligase and aminoacyl-tRNA amidotransferase. These changes suggest that related genes from transcription to translation in cotton seedlings under cold stress might be increased by treatment with EBR.

### 3.10 DEGs involved in protein processing in the endoplasmic reticulum (ER)

Cold, heat, salt, and drought stress can cause misfolded proteins that can be recognized by a quality control (QC) system and degraded by the ubiquitin-26S proteasome system [43]. Protein QC in the ER comprises many steps, including folding and transport of nascent proteins as well as degradation of misfolded proteins [44]. There were 49 gene homologs involved in protein processing in the ER (Table 3). Among them, the expression of 26 gene homologs was up-regulated, including protein transport protein Sec61, molecular chaperone HSP90, protein transport protein Sec61, mannosyl-oligosaccharide glucosidase (Glc1), heat shock protein (Hsp40), protein disulfide-isomerase (PDIs), alpha-mannosidase I (EDEM), mannosyl-oligosaccharide 1 (ERManI), calreticulin (CRM), and endoribonuclease IRE1. And the 23 down-regulated genes were mainly focused on ER-associated degradation (ERAD) and the ubiquitin ligase complex, including Hrd1, RMA1, UbcH5, CHIP and so on (Table 3). These results suggest that ribosome anchor (Glc1), protein recognition by luminal chaperones (Hsp40), and protein targeting degradation (PDIs) might be enhanced.

**Table 3:** DEGs involved in protein processing in the endoplasmic reticulum identified by comparing the Cold+water and Cold+EBR treatments.

Gene ID	log2(FC)	Pvalue	FDR	Symbol	Description
Gh_D05G0057	1.023662	0.001508	0.021673	SEC61	Protein transport protein SEC61 subunit alpha
Gh_D01G1589	1.591235	0.000406	0.008455	GlcI	Mannosyl-oligosaccharide glucosidase
Gh_A01G2121	1.561879	0.001472	0.021373	GlcI	Mannosyl-oligosaccharide glucosidase
Gh_D06G2388	1.022196	0.000709	0.012765	NEF	Hypoxia up-regulated 1
Gh_A09G1410	1.536372	0.00149	0.021529	Hsp40	Heat shock protein 40
Gh_D09G1415	1.428843	8.11E-05	0.002725	Hsp40	Heat shock protein 40
Gh_D03G0651	2.928917	0.000141	0.00408	CRT	Calreticulin-3
Gh_A02G1051	2.653712	1.72E-06	0.000168	CRT	Calreticulin-3
Gh_A10G0028	1.988917	0.000106	0.003303	ERManI	Mannosyl-oligosaccharide alpha-1,2-mannosidase
Gh_A02G0907	1.016023	0.002488	0.030809	ERManI	Mannosyl-oligosaccharide alpha-1,2-mannosidase
Gh_D09G1614	1.602884	0.000789	0.0138	EDEM	ER degradation enhancer, mannosidase alpha-like 1
Gh_A05G3724	1.107959	0.001897	0.025514	PDI	Protein disulfide-isomerase A1
Gh_D05G1614	1.335718	0.000371	0.007935	Derlin	Derlin-1
Gh_Sca013597G01	-11.0339	5.05E-08	1.19E-05	Hsp70	Heat shock protein 70
Gh_D06G1945	1.478498	0.00045	0.009145	Hsp40	Heat shock protein 40
Gh_D13G0091	1.382835	1.66E-05	0.000877	Hsp40	Heat shock protein 40
Gh_D12G0270	1.201387	0.000496	0.009845	Hsp40	Heat shock protein 40
Gh_Sca005047G01	-1.00364	0.000603	0.011378	Hsp40	Heat shock protein 40
Gh_D04G0018	1.296906	4.54E-07	6.02E-05	Hsp40	Heat shock protein 40
Gh_A02G1769	1.874469	0.000612	0.011491	Hsp40	Heat shock protein 40
Gh_D07G0512	2.02284	9.19E-05	0.003007	Hsp40	Heat shock protein 40
Gh_A07G0448	3.954196	0.002271	0.028892	Hsp40	Heat shock protein 40
Gh_D02G0860	-2.16727	0.000225	0.005664	Hsp40	Heat shock protein 40
Gh_A07G1723	1.339416	0.00349	0.039347	Hsp90	Heat shock protein 90
Gh_D01G0761	1.195052	4.05E-06	0.000307	Hsp90	Heat shock protein 90
Gh_D02G1319	1.066936	3.18E-05	0.001388	Hsp90	Heat shock protein 90
Gh_D13G0273	-1.23276	0.00065	0.01198	Ufd2	Ubiquitin conjugation factor E4 B
Gh_A13G0256	-1.62973	9.72E-07	0.000107	Ufd2	Ubiquitin conjugation factor E4 B
Gh_D11G1748	1.138211	0.001294	0.019501	ATF6	Cyclic AMP-dependent transcription factor ATF-6 alpha
Gh_A05G2508	-1.29521	0.000313	0.007145	ATF6	Cyclic AMP-dependent transcription factor ATF-6 alpha
Gh_D05G2786	-1.63366	1.37E-05	0.000764	ATF6	Cyclic AMP-dependent transcription factor ATF-6 alpha
Gh_Sca006599G01	-1.5898	0.000239	0.00591	ATF6	Cyclic AMP-dependent transcription factor ATF-6 alpha
Gh_A05G2447	-2.19465	0.000397	0.008324	ATF6	Cyclic AMP-dependent transcription factor ATF-6 alpha
Gh_A13G1065	1.562275	0.000133	0.003903	IRE1	Serine/threonine-protein kinase/endoribonuclease
Gh_A02G1715	-2.41423	9.94E-06	0.000606	IRE1	Serine/threonine-protein kinase/endoribonuclease
Gh_A01G1393	2.362323	0.000281	0.006603	UbcH5	Ubiquitin-conjugating enzyme E2 D
Gh_A03G2115	-2.05655	8.51E-07	9.82E-05	CHIP	STIP1 homology and U-box containing protein 1
Gh_A04G0474	-2.66626	0.001347	0.020063	CHIP	STIP1 homology and U-box containing protein 1
Gh_A04G0476	-1.98811	1.11E-05	0.000656	CHIP	STIP1 homology and U-box containing protein 1
Gh_A09G0943	-1.52738	0.001425	0.020879	CHIP	STIP1 homology and U-box containing protein 1
Gh_A11G3090	-1.03402	1.51E-05	0.000817	CHIP	STIP1 homology and U-box containing protein 1
Gh_D02G1367	-2.64765	6.86E-07	8.33E-05	CHIP	STIP1 homology and U-box containing protein 1
Gh_D04G0882	-5.07325	0.001479	0.021426	CHIP	STIP1 homology and U-box containing protein 1
Gh_D09G0975	-1.6477	0.003321	0.037958	CHIP	STIP1 homology and U-box containing protein 1
Gh_D11G1714	-1.07191	0.001005	0.016405	CHIP	STIP1 homology and U-box containing protein 1
Gh_D12G2576	-2.15645	0.001043	0.016816	CHIP	STIP1 homology and U-box containing protein 1
Gh_A12G0616	-1.05147	0.002882	0.034075	RMA1	E3 ubiquitin-protein ligase
Gh_D09G1011	-2.13355	5.57E-09	2.28E-06	Hrd1	E3 ubiquitin-protein ligase synoviolin
Gh_D13G2088	-1.07468	0.000213	0.005442	Hrd1	E3 ubiquitin-protein ligase synoviolin

### 3.11 EBR down-regulates expression of abscisic acid and ethylene signal transduction-related genes

**Fig 8:**
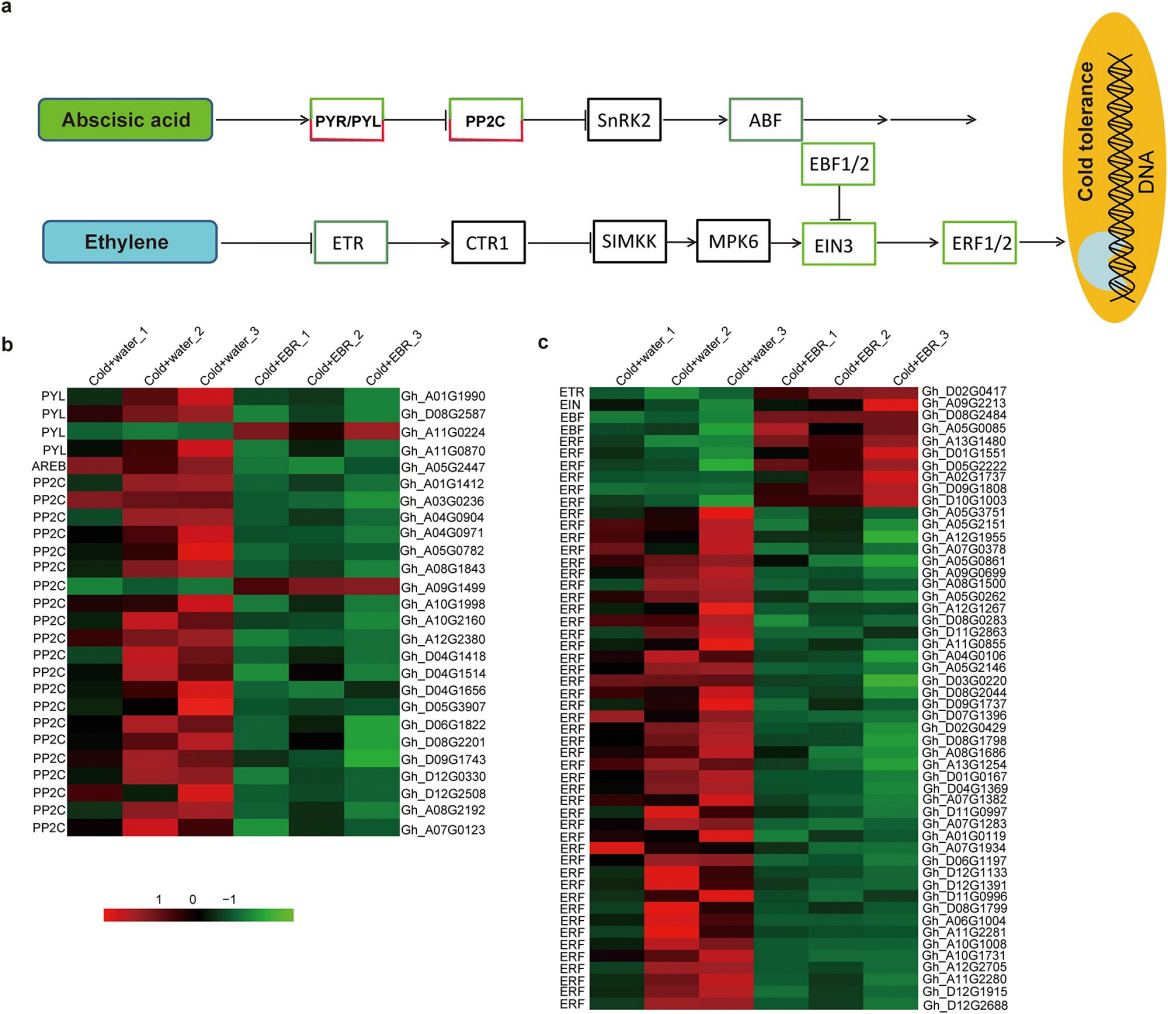
Expression patterns of ABA and ETH signal transduction-related genes. **a**. ABA and ETH hormone signal transduction. Green rectangles indicate down-regulated genes. Red rectangles indicate up-regulated genes. Black rectangles indicate that these pathway genes were not found. Arrows and inhibition lines represent activation and suppression, respectively. **b and c**. Expression patterns of ABA (b) and ETH (c) hormone signal transduction-related genes in the three replicates of the Cold+water and Cold+EBR treatments. The colored boxes show the relative gene expression levels from low (green) to high (red).

Hormones play an important role in plant growth, development, and adaptation to adversity. Ethylene and abscisic acid act as negative regulators in regulating plant growth promotion and plant senescence, and are also known as stress hormones [45]. The KEGG pathway annotation of the DEGs showed that the down-regulated DEGs are significantly enriched in plant hormone signal transduction (ko04075) (Fig 8a). Based on the KEGG pathway of ko04075, the DEGs were mainly enriched in the ABA and ETH signal transduction pathways.

All the ETH biosynthesis- and signal transduction-related genes showed down-regulated expression, including genes for 1-aminocyclopropane-1-carboxylate synthase (ACS), an enzyme that catalyzes the rate-limiting step in ETH biosynthesis, the ETH receptor (ETR), ETH insensitive (EIN), and most of the ETH-responsive transcription factors (ERFs) (Fig 8b, S6 Table). EBR treatment under cold stress downregulated the expression of the gene encoding zerumbone synthase (ZSD), an enzyme involved in the conversion of xanthoxin to ABA-aldehyde during ABA biosynthesis. Furthermore, expression of ABA signal transduction-related genes was also decreased, including genes that encode the ABA receptor (PYL), and protein phosphatase 2C (PP2C) which is involved in ABA signal transduction and negatively regulates the ABA response (Fig 8c, S6 Table). ABA binds to the receptor PYL, changing the conformation of the receptor, which then interacts with PP2C phosphatase family members to inhibit PP2C activity, release CIPK activity, and phosphorylate related transcription factors, thereby initiating the expression of ABA-responsive genes [46]. Both ABA and ETH are stress hormones, the EBR treatment down-regulated expression of ETH- and ABA-related signal transduction related genes, which indicates that EBR increases cold tolerance in cotton seedlings by inhibiting ABA and ETH signal transduction.

## 4. Discussion

Cotton is one of the most important economic crops in China; and the autonomous region of Xinjiang is the main cotton-producing area in China. Cold stress, especially a late frost during spring, can negatively affect cotton seedling development and can strongly limit cotton production and quality in Xinjiang. BRs, a class of steroidal hormones, participate widely in plant growth, development, photomorphogenesis, and response to adversity [47]. Studies in peppers [48], perennial ryegrass [15] and Arabidopsis [49] have shown that BR can increase plant tolerance to cold stress. Therefore, we measured physiological characters and performed RNA-sequencing to illustrate BR functions in cotton cold tolerance.

### 4.1 EBR enhances cold tolerance in cotton seedlings in a dose-dependent manner

In plants, cold stress primarily disrupts the plasma membrane and induces membrane permeability, which increases MDA content and ion leakage [50, 51]. Under conditions of cold stress, cotton seedlings were treated with a concentration range of EBR (0, 0.05, 0.1, 0.2, 0.5, and 1.0 mg/L) which alleviated the MDA content and ion leakage. Also GO and KEGG enrichment analysis showed that the DEGs were enriched in genes involved in antioxidant-related glutathione metabolism processes. Under cold stress, plant cells accumulate proline to protect against protein denaturation [52]; in cotton seedlings treated with EBR, the proline content was higher than in the control and the plants had greater cold tolerance. The cotton seedlings treated with EBR under cold stress had higher Pn. Also, photosynthesis-related DEGs were up-regulated based on the RNA-seq data. In this study, a range of EBR concentrations were used to treat cotton seedlings; the physiological index showed that 0.2 mg/L is the optimal concentration for increasing cold tolerance in cotton seedlings.

### 4.2 EBR is involved in the biosynthesis of gossypol and glutathione metabolism in cotton cold tolerance

According to previous findings, plants under adverse environmental conditions usually accumulate secondary metabolites that activate defense mechanisms to acclimate against stresses [53]. Under cold stress, the levels of sesquiterpenes in *Artemisia annua* [54], anthocyanins in *Nicotiana tabacum* [55], quercetin in *Arnica montana* L [56], and phloroglucinols in *Hypericum* spp. [57] were increased under cold stress. Therefore, it is reasonable that both up- and down-regulated genes are involved in the biosynthesis of secondary metabolites. Gossypol is one of the most important secondary metabolites in cotton, and there were down-regulated DEGs encoding enzymes that catalyze four successive steps in gossypol biosynthesis in cotton cotyledons under cold stress (Fig 5). Therefore, we speculated that EBR is involved in the biosynthesis of secondary metabolites, especially gossypol biosynthesis as it relates to cold tolerance in cotton.

Stress often changes the redox homeostasis on both sides of the plasma membrane by inducing an oxidative burst of reactive oxygen species (ROS) on the plasma membrane. There are two small organic molecules in plants that are mainly involved in regulating the dynamic balance of redox homeostasis inside and outside the plasma membrane: glutathione and ascorbic acid [58]. Therefore, ascorbic acid and glutathione are the main molecules that resist oxidative stress and maintain the dynamic equilibrium of redox in plastids. The DEGs were significantly enriched in glutathione metabolism, and two genes for glutathione metabolism-related enzymes were found; glutathione peroxidase (GPX) and glutathione S-transferase (GST). Genes encoding the ascorbic acid metabolism-related enzymes L-ascorbate oxidase (ASO) and dehydroascorbate reductase (DHAR) were up-regulated by EBR.

### 4.3 Exogenous application of EBR promotes genetic information processing under cold stress

Proteins are the executors of gene function. The process of going from DNA to mRNA to protein involves a series of complex expression regulation mechanisms, including transcriptional regulation, post-transcriptional regulation, translation regulation, and post-translational regulation. Both GO and KEGG enrichment analyses showed that of the DEGs participated in the processes of DNA to protein including transcription, translation, protein folding, sorting, and degradation pathways.

Proteins enter the ER soon after protein synthesis begins on the ribosomes, which continue to synthesize peptide chains and their modifications in the endoplasmic reticulum. N-glycosylation is one of the most important protein modifications; studies have shown that the degradation of cold-regulated glycoproteins increases in response to cold stress in Arabidopsis [59]. The N-glycan biosynthesis KEGG pathway was significantly enriched, but N-glycan is not the only raw material for protein N-glycosylation that can enhance glycoprotein tolerance to cold stress. N-glycan is also a soluble sugar that increases the cellular solute concentration to decrease protein denaturation as the proline content increases in response to cold stress.

In the endoplasmic reticulum, there is a system specifically responsible for detecting protein folding called the endoplasmic reticulum quality control system (ERQC). Degradation of misfolded proteins and cold-regulated glycoproteins [49], through polyubiquitination and degradation in the ubiquitin-proteasome system, is one of the most important plant responses to mitigate the damage caused by the accumulation of misfolded proteins under environmental stresses such as cold, heat, drought, salinity, and infections by bacterial and viral pathogens [60]. Timely removal of abnormal and denatured proteins is crucial for plant development and the stress response [61]. A ubiquitin gene from wheat, *TaUB2*, improved tolerance to cold, salt, and drought when overexpressed in tobacco [61, 62]. The up-regulated DEGs were significantly enriched in the ubiquitin-mediated proteolysis pathway, which indicates that EBR treatment improved cellular ubiquitin mediated proteolysis to protect cotton seedlings from cold stress injury.

### 4.4 EBR increases cold tolerance in cotton seedlings by down-regulating ETH and ABA signal transduction

Plant hormones are low molecular weight compounds that signal information from a synthesis, catabolism, and transport response to cold stress [63]. Knowledge of the molecular mode of action of BRs remains patchy because of cross-talk with signaling cascades conferring tolerance to other environmental stimuli, and also because the hormone modes of action vary between plant species [64]. When treated with EBR under cold stress conditions, ETH and ABA biosynthesis and signal transduction were suppressed. Studies on chilling stress in pepper showed that treatment with EBR could increase the endogenous IAA content and suppress the ETH content [48]. ABA antagonizes the promotive effect of BR on leaf angle [65]. Low temperature can promote the synthesis of ethylene and also ethylene-induced membrane permeability [66]. In cotton seedlings, EBR treatment reduced MDA content and electrolyte leakage, which indicates that BR and ETH have an antagonistic effect on cold stress in cotton seedlings. Above all, EBR increases cold tolerance in cotton seedlings by down-regulating ETH and ABA signal transduction.

## Supporting information

S1 Table GO analysis of the DEGs regulated by the Cold+water and Cold+EBR treatments. (XLS)

S2 Table KEGG pathway enrichment of DEGs up-regulated by the Cold+water and Cold+EBR treatments. (XLSX)

S3 Table KEGG pathway enrichment of DEGs down-regulated by the Cold+water and Cold+EBR treatments. (XLSX)

S4 Table Glutathione metabolism-related DEGs in the Cold+water and Cold+EBR treatments. (XLSX)

S5 Table Transcription and translation process-related DEGs in the Cold+water and Cold+EBR treatments. (XLSX)

S6 Table ABA and ETH signal transduction-related DEGs in the Cold+water and Cold+EBR treatments. (XLSX)
